# Characteristics and Influencing Factors on the Hollowing of Traditional Villages—Taking 2645 Villages from the Chinese Traditional Village Catalogue (Batch 5) as an Example

**DOI:** 10.3390/ijerph182312759

**Published:** 2021-12-03

**Authors:** Chunla Liu, Mei Xu

**Affiliations:** 1 Key Laboratory of Geospatial Big Data Mining and Application, College of Geographic Sciences, Hunan Normal University, Changsha 410081, China; 2 College of Tourism, Central South University of Forestry and Technology, Changsha 410004, China; T20142195@csuft.edu.cn

**Keywords:** hollowing, traditional villages, characteristics, influencing factors, China

## Abstract

With the rapid development of urbanization and modernization, the population of traditional villages migrates into surrounding areas, causing the hollowing of traditional villages. The disintegration of China’s traditional village means the loss of historical memory and cultural characteristics of ethnic regions, seriously endangering the country’s cultural heritage. To better understand the hollowing phenomenon, this study analyzed 2645 villages from the Chinese traditional village catalogue (Batch 5) and evaluated different village attributes, including location, household registration, permanent population, number of traditional buildings, cultural relics, historical buildings, and non-heritage representative projects. We constructed an evaluation index system and used the entropy weight method, comprehensive evaluation method, and correlation analysis method to quantitatively assess the characteristics and influencing factors of hollowing among traditional Chinese villages. The main results are as follows: ① The hollowing index was above 0.5; most traditional villages have entered the stage of high hollowing. ② The traditional villages with hollowing index above 0.9 comprised 92%, and those between 0.8 and 0.9 made up 6%. Those with hollowing index at intervals 0.7–0.8, 0.6–0.7, and 0.5–0.6 accounted for 0.98%, 0.30%, and 0.11%, respectively. ③ Population hollowing is the fundamental cause of traditional village hollowing. In more than 99% of traditional villages, the population hollowing index was greater than 0.7. ④ More than 99% of traditional villages have a building hollowing index greater than 0.4, and more than 92% of the villages had a per capita number below 0.1. ⑤ The cultural hollowing rate for most traditional villages was very high. The cultural hollowing index for more than 99% of traditional villages was greater than 0.7. This study provides references for government administrators and scholars in rural revitalization and traditional village hollowing governance.

## 1. Introduction

The traditional village is a long-standing form of rural settlement and the largest heritage left by agricultural civilization. It not only functions as a historical and cultural heritage but also provides important value for promoting the construction of ecological civilization, condensing historical memory, and reflecting the progress of civilization [1,2,3,4,5]. With rapid urban development and modernization, rural populations have continued to migrate into urban centers or neighboring villages, resulting in the hollowing of traditional villages [6,7,8]. While the phenomenon of traditional village hollowing occurs worldwide [9,10], the current situation in China is far more complicated and more serious than that in other countries [11,12]. To a large extent, China’s traditional village is a unique spatial form of rural settlement shaped by the special social system of the country’s urban-rural dual structure, which varies significantly from the evolution and development of foreign villages [11,13,14]. The hollowing of traditional villages threatens the loss of invaluable national historical memory and heritage and the regional cultural characteristics of ethnic groups, seriously endangering village heritage and cultural inheritance [5,15]. Therefore, traditional village hollowing has become a major policy, administrative, and research issue in human and economic geography. 

In October 2017, the Rural Revitalization Strategy was put forward in the 19th National Congress of the Communist Party of China. In 2018, the Central Document No. 1 “Opinions of the CPC Central Committee and the State Council on the Implementation of the Rural Revitalization Strategy” was released. The report explicitly identified the requirements for the protection of traditional villages, marking the significant transformation of the development of traditional villages (an important rural settlement carrier that highlights and inherits excellent traditional Chinese culture). The hollowing management of traditional villages is an important aspect of Rural Revitalization. From research on traditional village hollowing in China, a series of important theoretical achievements have been made, learning Western ideas and combining them with practices of typical traditional villages in China. Different issues have been identified and explored, such as traditional village hollowing and over-commercialization [16], traditional village hollowing and its causes and protection ways [17], location differentiation characteristics of traditional village hollowing [18], population hollowing and settlement hollowing of the traditional village [19], and the formation mechanism of traditional village hollowing and governance policies [20,21]. On the whole, most existing studies are mesoscopic (regional) or microscopic (village) in scale and are usually analyzing only one particular aspect of traditional village hollowing. Comparative analyses on large-scale traditional village hollowing in China are still rare [12,14,22,23,24].

Research on the regional characteristics and influencing factors of traditional village hollowing helps understand the evolutionary characteristics in different regions and provides a useful reference on appropriate governance [25,26,27,28]. However, there is very little systematic research exploring the provincial differences of Chinese traditional village hollowing. This paper studies the provincial differences of Chinese traditional village hollowing from the perspectives of population hollowing, cultural hollowing, and building hollowing, discusses the influencing factors, and puts forward some relevant suggestions.

Determining how to best study the characteristics and influencing factors on the hollowing of traditional villages is an important issue. Unlike qualitative analysis, quantitative studies on traditional village hollowing have been limited. The most common techniques used in the quantitative examination of traditional village hollowing are the entropy-AHP model, push–pull model, questionnaire survey, logistic regression, multiple regression analysis, quantile regression model, PSR model, and geographically weighted regression (GWR) [18,25,29,30,31,32,33].

What are the characteristics of the hollowing of traditional villages? What are the influencing factors? How to find them? To probe these questions, we conducted this study. The single traditional village is used as the basic unit in this study. Using three subsystems—population hollowing, building hollowing, and culture hollowing—we comprehensively evaluated the spatial characteristics of traditional village hollowing and analyzed the provincial differences.

This study has important theoretical and practical significance. It enriches the theoretical research and case study on rural development in modern rural geography, developing methods for evaluating the characteristics of traditional villages. It also provides practical support to better understand the development status of Chinese traditional villages and to help optimize management policies.

## 2. Connotation of Traditional Villages Hollowing

Generally speaking, the hollowing of traditional villages results from the abandonment of traditional buildings, the outflow of population, and the expansion of the original villages. Its landscape features can be summarized as follows: a sharp decrease in population density, an increase in idle traditional buildings, the expansion of ruins, the decline of the originality of villages, and the sharp contrast with new expansion zones. Previous studies have pointed out that the hollowing of villages is a relative and fuzzy concept, a process where the original villages with relatively homogeneous aging develop into dual structures. Objectively, there is no absolute demarcation between the new expansion zone and the hollowing zone, which is embodied in the hollowing of population, industry, facilities, homestead, traditional buildings, and functional spaces [5,9,15,19,22,29,34,35].

**Figure 1:**
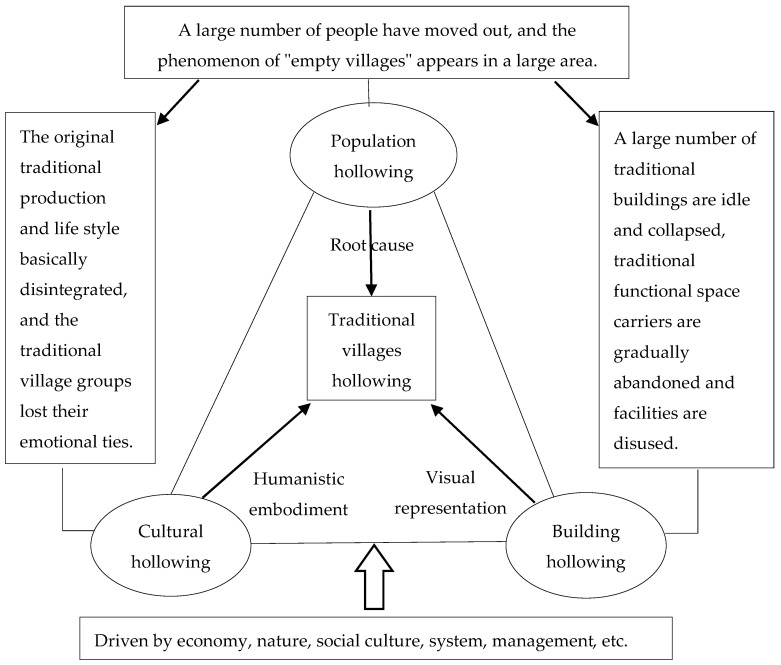
Connotation and basic framework of traditional villages hollowing. Note: Using expert consultation and the Edraw Max software, we developed the connotation and basic framework of traditional village hollowing.

**Figure 2:**
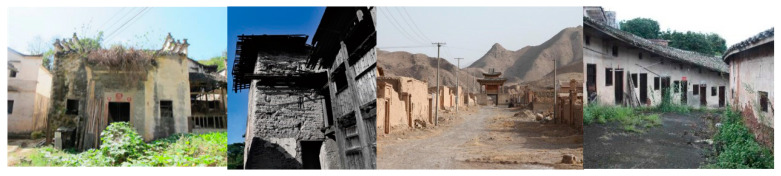
Representative traditional architectural landscape still exists in traditional villages. Note: From left to right: Zhuangyuan village, Huangshan City, Anhui Province; Zhaili Village, Fuzhou City, Fujian Province; Xiakou Village, Zhangye City, Gansu Province; Yaomei Village, Meizhou City, Guangdong Province.

Traditional village hollowing is a special human–environment relationship [24,36,37]. It reflects the disintegration of the human–environment relationship in rural areas, highlighted by the outflow of large populations, idleness of traditional buildings, and the lack of traditional culture inheritance. From the human–environment perspective [38,39,40], which emphasizes the interaction between human (e.g., population, culture) and environmental (e.g., natural environmental) factors, core traditional village hollowing can be summarized into the hollowing of three aspects: population, building, and culture. The other aspects of hollowing, including industry, homestead, traditional buildings, and functional spaces, are extensions of the three main aspects (see Table 1, Figure 1 and Figure 2).

(1) Population hollowing is an anthropogenic facet of traditional village hollowing. Due to geographic (i.e., remote location) and socio-economic (i.e., limited employment opportunities, underdeveloped infrastructure, poor living standards, and low access to cultural and entertainment facilities) reasons, many young and middle-aged residents have left the traditional villages, seeking better opportunities elsewhere [41,42,43]. As people emigrate, the permanent population is sharply reduced. With the outflow of cultural heritage inheritors and young and middle-aged labor force, fewer people inhabit traditional houses, develop rural production, and inherit the traditional village culture. This then results in industrial hollowing and homestead hollowing [23,32].

(2) Building hollowing is a visual representation of the hollowing of traditional villages. Due to natural factors (e.g., natural wear and tear) and anthropogenic factors (e.g., accelerated levels of urbanization and modernization), many cultural and historical buildings have collapsed or are significantly damaged [11,44,45]. Due to the decrease in village population and increasing inactivity, the phenomenon “empty houses, empty roads, empty villages” develops, as reflected in the material landscape of many uninhabited houses, overgrown weeds, and dilapidated structures. The hollowing of buildings further leads to the hollowing of homesteads, traditional buildings, and functional spaces [31,46,47,48].

(3) Cultural hollowing is the humanistic embodiment of the hollowing of traditional villages. The traditional village is an important carrier of traditional culture inheritance. The people who stay in traditional villages are mostly the elderly and children, who are unable to pass on the inheritance of the intangible cultural heritage [15,19,49]. Due to the migration of people and degradation of vacant facilities, the traditional lifestyle and spiritual spaces are gradually forgotten and will inevitably disappear. Generally speaking, the hollowing of the population largely determines the main behavior of hollowing land use in traditional villages. The hollowing of buildings and culture greatly affects governance and resource utilization [22,50,51,52,53,54].

**Table 1:** Contrast between normal village hollowing and traditional village hollowing.

Contrastive Items	Normal Villages Hollowing	Traditional Villages Hollowing
Population hollowing	Loss of young and middle-aged labor	With the loss of the young and middle-aged labor force, traditional culture faces the situation of having no successors.
Building hollowing	Empty houses, new buildings constructed, and the old ones not demolished	On the one hand, there is a phenomenon of “empty houses, new buildings constructed and the old ones not demolished”; meanwhile, traditional buildings and historical buildings will be left unattended and collapse one after another, and historical and cultural carriers will soon die out.
Cultural hollowing	Depressed villages without vitality	Villages are depressed and lack vitality, traditional culture, historical memory.

Note: There are some differences between historical buildings and traditional buildings. Historical buildings refer to the buildings with certain protection value that is determined and announced by the people’s governments of cities and counties. These buildings can reflect the historical features and local characteristics. They are different from cultural relics, protection units, and buildings or constructions of immovable cultural relics. Cultural relics buildings have certain cultural value, while historical buildings have certain construction value. The concept of traditional buildings is one of time category. For example, Chinese traditional buildings refer to the buildings from Pre-Qin to the mid-19th century, an independent building system.

## 3. Research Methods and Data Sources

**Figure 3:**
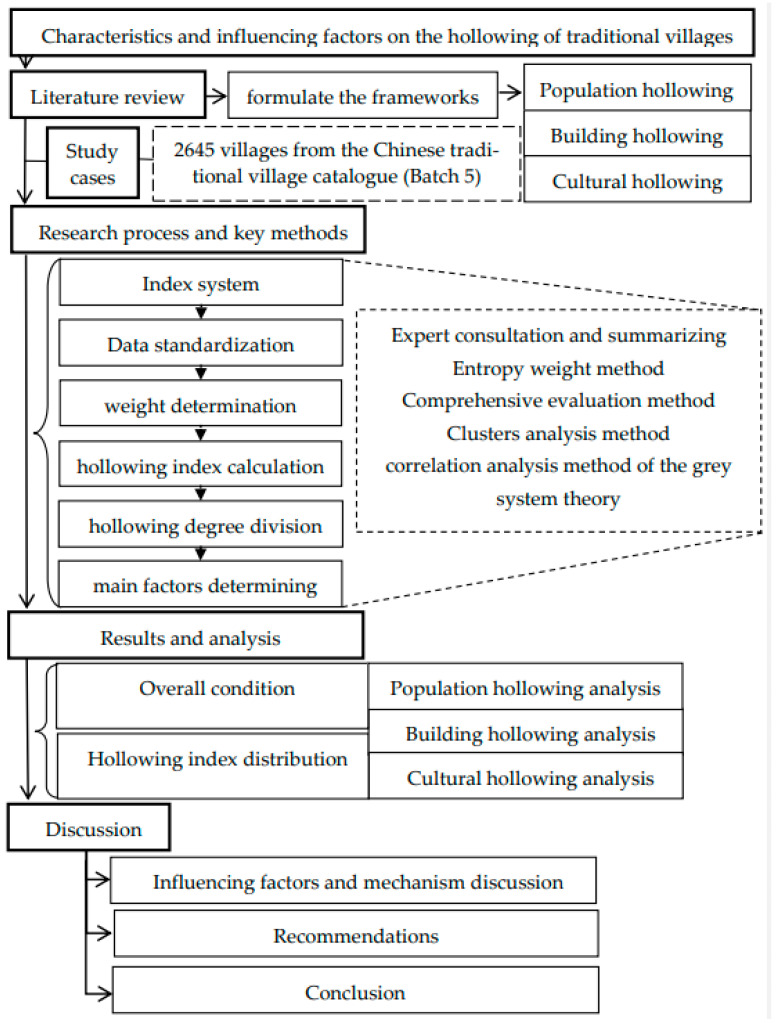
Flow chart diagram of the research process and key methods.

After constructing the evaluation index system for traditional village hollowing, we explored the characteristics of the hollowing of 2645 villages from the Chinese traditional village catalogue (Batch 5) (In China, 646 villages were included in the first batch (batch 1) of the Chinese traditional village catalogue in 2012, the second batch (batch 2) was in 2013, the third batch (batch 3) was in 2014, the fourth batch (batch 4) was in 2016, and the fifth batch (batch 5) was in 2019). The dataset was first standardized, and the entropy weight method was used for weight determination. A comprehensive evaluation method was adopted to calculate the hollowing index, and the calculation results of the hollowing degree were combined and categorized into clusters. The grey system theory’s correlation analysis method was then used to determine the main factors affecting the hollowing of traditional villages, which were then used in developing the policy recommendations (see Figure 3).

### 3.1. Index System

In the evaluation of village hollowing, indicators reflecting the vacancy state are generally used, such as the vacancy rate of residential buildings. However, the hollowing degree of traditional Chinese villages is extremely serious and extensive, and many traditional buildings are almost completely empty (especially historical buildings) [16,17,18,19,20,21,50]. If we continue analyzing the hollowing of traditional Chinese villages in highly general terms, it would be difficult to identify regional differences of hollowing from the macro scale. Therefore, the hollowing of traditional villages should not be simply evaluated using traditional evaluation methods of rural development.

Given the diversity and complexity of factors affecting the hollowing of traditional Chinese villages, this paper explored the prevailing conditions of 2645 traditional villages. These villages are part of the fifth batch of the Chinese traditional village catalogue released by the Ministry of Housing and Urban-Rural Development of the People’s Republic of China (MOHURD). Using the current evaluation index system and recommendations from existing research [23,32,33,41,44,46,47], five indicators were selected based on the principle of “seeing people, things and life” (see Table 2).

(1) According to the Lewis model and the Petty–Clark theorem, with the developments of the society and the economy, the labor force tends to flow from the countryside into the city and from primary industry into secondary or tertiary industries. For traditional villages, as labor flows out, the resident populations decline, and the hollowing conditions become more severe. In many studies, the proportion of the resident population to the total registered population is usually calculated [32,44]. The smaller the ratio, the higher the hollowing degree of the population.
(2) Houses become empty as people move out [30,42,48]. Indexes, such as homestead vacancy rate, are commonly calculated to indicate building hollowing [33]. The number of traditional and historical buildings can be used as a metric for traditional village hollowing. Given the difficulty of calculating the area of traditional villages and building vacancy rates at the national level, two indicators are used, based on the data released by the MOHURD: the number of traditional buildings per capita and the number of historical buildings per capita.
(3) Cultural hollowing mainly refers to the hollowing of traditional culture and its carrier caused by the loss of population and the destruction of hardware facilities in traditional villages [31,51]. Given the difficulty of calculating traditional village culture at the national level, the data released by the MOHURD can be used to calculate the number of cultural relics per capita and the number of representative intangible cultural heritage projects per capita. The indicators “proportion value” and “per capita value” are used to reflect the overall situation of traditional villages hollowing.

**Table 2:** Evaluation index system of traditional villages hollowing.

	Evaluation Index		Calculation Method	Remarks
	Primary Index	Secondary Index		
Evaluation of hollowing degree of traditional villages	Population hollowing	The proportion of resident population, X1	Resident population/Total registered population	-
	Building hollowing	Traditional buildings per capita, X2	Traditional buildings/Total registered population	-
		Historical buildings per capita, X3	Historical buildings/Total registered population	-
	Cultural hollowing	The number of cultural relics per capita, X4	The number of cultural relics/Total registered population	-
		The number of representative intangible cultural heritage projects per capita, X5	The number of representative intangible cultural heritage projects/Total registered population	-

Note: “-” in the “remarks” column indicates that the index has a negative effect on the evaluation target.

### 3.2. Research Methods

(1) Data standardization

Since some research index data are not suitable for comparative analysis, the dataset must be standardized first [55,56,57]. The metrics are negative indicators, which are calculated using the formula

X_i_* = (X_max_ − X_i_)/(X_max_ − X_min_) (1)

where X is the sample matrix of traditional village hollowing index corresponding to n samples and m evaluation indexes, X = (X_ij_)_m×n_; X_i_* is the standardized value of each element; X_i_ is the initial data of each element; and X_max_ and X_min_ are the maximum and minimum values, respectively.

(2) Entropy weight method for weight determination

The entropy weight method can objectively determine the weight of each factor. The entropy weight method is used for calculation [55,56], using the expression

(2) $$W_{i}=\frac{1-H_{i}}{m-\sum_{i=1}^{m}H_{i}}$$

such that

(3) $$H_{i}=-K\sum_{j=1}^{n}f_{\mathrm{ij}}\ln f_{\mathrm{ij}}$$

(4) $$f_{ij}=\frac{r_{ij}}{\sum_{j=1}^{n}r_{\mathrm{ij}}}$$

(5) $$k=\frac{1}{\ln N}$$

(6) $$r_{\mathrm{ij}}=\frac{\max\left|x_{\mathrm{ij}}\right|{-x}_{\mathrm{ij}}}{\max\left|x_{\mathrm{ij}}\right|-\min\left|x_{\mathrm{ij}}\right|}\times 10$$

N = 1, 2, …, n, i = 1,2, …, m; j = 1, 2, …, n.

In the formula, W_i_ is the weight value of the ith factor, r_ij_ is the data matrix (extremum method), H_i_ is the entropy of the ith index. When f_ij_ = 0, then lnf_ij_ = 0. Based on the formula, the smaller the information entropy H of an index, the greater the variation degree of its index value, and the more information it provides. This means that the said factor plays a greater role in the comprehensive evaluation and is therefore given greater weight (see Table 3).

(3) Calculation of the hollowing index and division of hollowing degree

The comprehensive evaluation method is adopted for calculation [56,57], and the evaluation model is as follows:

(7) $$Y=\sum_{i=1}^{m}W_{i}F_{i}$$

where Y is the hollowing index of traditional villages, W_i_ is the index weight, and F_i_ is the corresponding index value.

The calculation results for population hollowing, building hollowing, and cultural hollowing are combined and categorized into clusters [44,52,53]. When y ≥ 0.85, the traditional villages are in a high hollowing state. When 0.50 ≤ y < 0.85, the traditional villages are in a moderate hollowing state. When 0.20 ≤ y < 0.50, the traditional villages are in a potential hollowing state. When y < 0.20, the traditional villages are in a low hollowing state.

(4) Determining the main factors affecting the hollowing of traditional villages

The correlation analysis method (Pearson method) of the grey system theory is used to reflect the degree of relationship between variables, given by the expression

(8) $$\varepsilon_{i}(j)=\frac{\underset{i}{\min}\underset{j}{\min}\left|X_{0}{(j)-X}_{i}(j)\right|+\mu \underset{i}{\max}\underset{j}{\max}\left|X_{0}{(j)-X}_{i}(j)\right|}{\left|X_{0}{(j)-X}_{i}(j)\right|+\mu \underset{i}{\max}\underset{j}{\max}\left|X_{0}{(j)-X}_{i}(j)\right|}$$

where j is the sample, j = 1, 2, …, n; i is the index code, and μ is the resolution coefficient. In general, μ∈[0, 1]; $\varepsilon_{i}(j)$ is the correlation coefficient of the relative reference number $x_{i}$. In this paper, μ is taken as 0.5.

By calculating the correlation degree, we can determine the impact of sub-indicators on the hollowing of traditional villages. The model is as follows [56]:

(9) $$r_{i}=\frac{1}{n}\sum_{j=1}^{n}\varepsilon_{i}\left(j\right)$$

where $r_{i}$ is the correlation degree of indicator i to hollowing of traditional villages. The larger the correlation degree, the more the indicator becomes the focus of traditional village hollowing.

### 3.3. Data Sources

The Batch 5 data were produced and extracted in 2019. Released by the MOHURD, the dataset was composed of 2645 villages (There are 2646 villages in the “fifth batch of villages to be listed in the Chinese traditional villages catalogue”, but Shuangfang village, Shaoyuan Town, Jiyuan City, Henan Province, has no relevant text and data materials. In order to ensure the unity of data, it is not included in the total number of samples in this study.) The data included the following information: village name, village attribute, location, household, registration population, permanent population, number of traditional buildings, cultural relics, historical buildings, intangible cultural heritage representative projects, titles and profiles of villages, and other basic data and information. The information database of traditional Chinese villages was constructed with 2645 (rows) and 12 (columns).

## 4. Results and Analysis

### 4.1. Overall Condition

**Figure 4:**
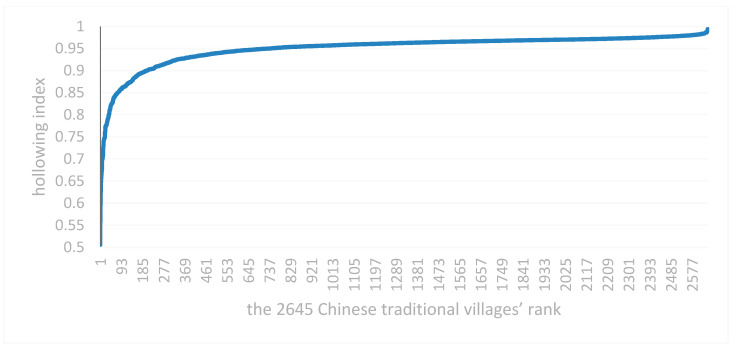
Overall distribution situation of Chinese traditional villages hollowing index. Note: The 2645 Chinese traditional village ranking was based on the village’s hollowing index.

**Figure 5:**
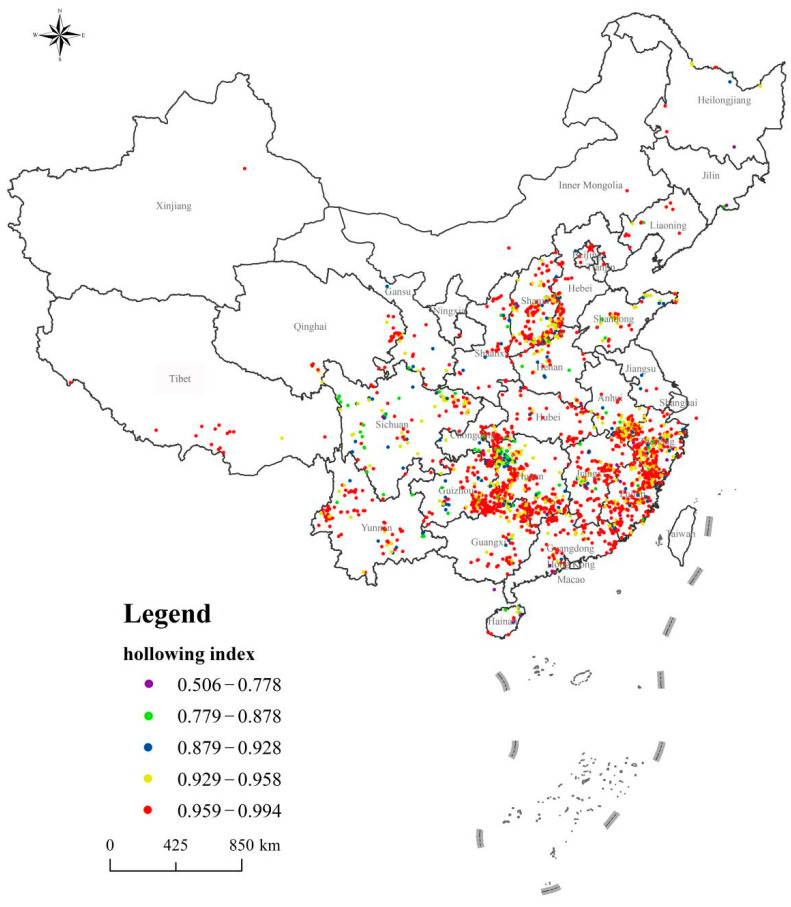
Distribution of Chinese traditional villages hollowing index.

The hollowing indices for the 2645 traditional Chinese villages were all above 0.5. About 92% of the traditional villages had a hollowing index greater than 0.9 and were mainly distributed in the provinces of Anhui, Beijing, Fujian, Gansu, Guangdong, Guangxi, Guizhou, Hainan, Hebei, Henan, Heilongjiang, Hubei, Hunan, Jilin, Jiangsu, Jiangxi, Liaoning, Inner Mongolia, Ningxia, Qinghai, Shandong, Shanxi, Shaanxi, Sichuan, Tianjin, Tibet, Xinjiang, Yunnan, Zhejiang, and Chongqing. Most of the traditional villages have entered the stage of high hollowing (see Figure 4 and Figure 5). The mean value of the comprehensive hollowing coefficient was 0.950, the median was 0.962, the standard deviation was 0.040, and the variance was 0.002. Based on the calculated correlation degrees (the *r*-value is 0.67, and the *p*-value < 0.05)), the primary factor for traditional village hollowing was building hollowing (correlation coefficient = 0.43, correlation degree = 0.86), followed by population hollowing (correlation coefficient = 0.30,correlation degree = 0.61) and cultural hollowing (correlation coefficient = 0.27, correlation degree = 0.63).

With the rapid socio-economic development urbanization, the rural populations have been migrating into cities and towns, and many traditional village populations have seen significant declines. With the large emigration of heritage inheritors and young workforce, various local aspects of traditional culture and customs are not effectively passed on, fundamentally disintegrating some cultural features and causing the hollowing of traditional villages to be more pronounced.

At the same time, the urban culture brought by those living outside permeates gradually into traditional villages, chipping away some aspects of traditional village living. Traditional buildings are gradually removed and replaced, and the village architectural landscape is seriously damaged. With the hollowing of population, culture, and buildings, traditional villages gradually enter the hollowing stage.

### 4.2. Hollowing Index Distribution

Traditional villages with a hollowing index greater than 0.9 were distributed in all provinces. Traditional villages with hollowing index greater than 0.8 and less than 0.9 accounted for 6.31% and were mainly distributed in the provinces of Anhui, Fujian, Guangdong, Guangxi, Guizhou, Hainan, Hebei, Henan, Hubei, Hunan, Jilin, Jiangxi, Liaoning, Shandong, Shanxi, Shaanxi, Sichuan, Yunnan, Zhejiang, and Chongqing. Traditional villages with hollowing index greater than 0.7 and less than 0.8 accounted for 0.98% and were mainly distributed in Anhui, Fujian, Gansu, Guangdong, Guangxi, Guizhou, Hainan, Henan, Heilongjiang, Hunan, Jilin, Jiangxi, Liaoning, Shandong, Shanxi, Sichuan, Zhejiang, and Chongqing. Traditional villages with hollowing indexes greater than 0.6 and less than 0.7 accounted for 0.30% and were found in Fujian, Guangdong, Guangxi, Henan, Shanxi, and Zhejiang. Traditional villages with a hollowing index greater than 0.5 and less than 0.6 accounted for 0.11% and were mainly distributed in Hainan, Henan, and Zhejiang (see Table 4).

### 4.3. Population Hollowing

**Figure 6:**
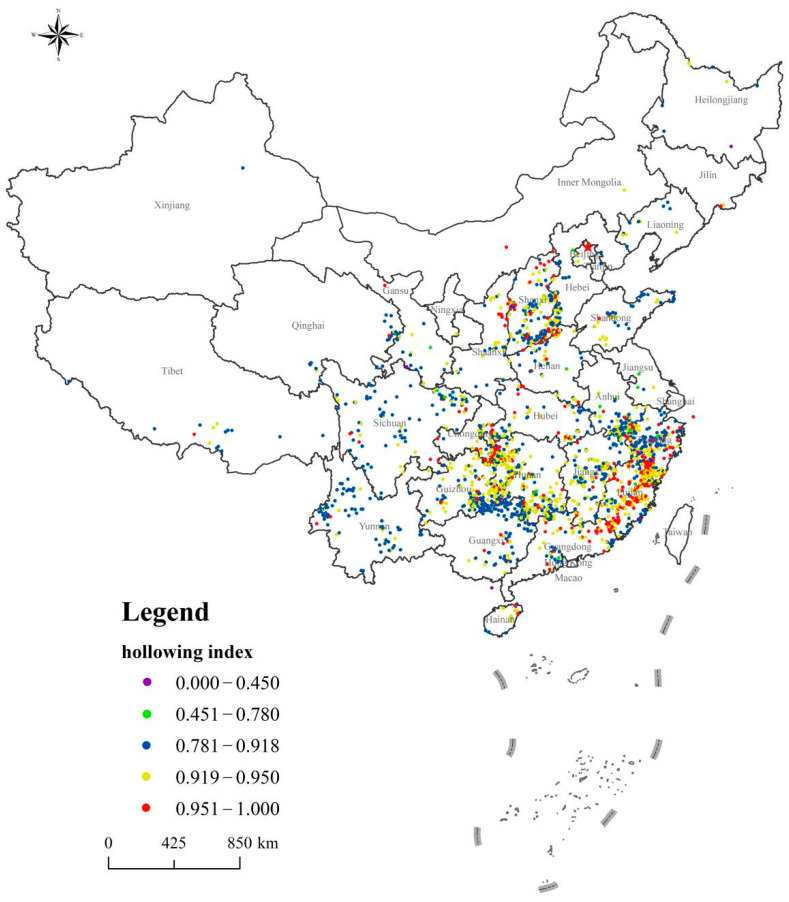
Distribution of Chinese traditional villages population hollowing index.

Population hollowing is the fundamental cause of traditional village hollowing. The mean value of the population hollowing coefficient for the 2645 villages was 0.920. The median was 0.920, the standard deviation was 0.054, the variance was 0.003, and the mode was 0.903 (the frequency of occurrence was 10%). In general, the population hollowing rate for most traditional villages was very high, and the population hollowing index of more than 99% of traditional villages was greater than 0.7 (see Figure 6). Most traditional villages were remote, economically backward, and had few employment channels. Most of the young and middle-aged laborers have opted to leave to make a living and work. There were also facets of traditional villages that could have promoted population hollowing, such as inconvenient transportation, poor living standards, low-quality education and health conditions, and low access to cultural and entertainment facilities. These issues force economically capable families to move out, further exacerbating the population hollowing in traditional villages.

### 4.4. Building Hollowing

**Figure 7:**
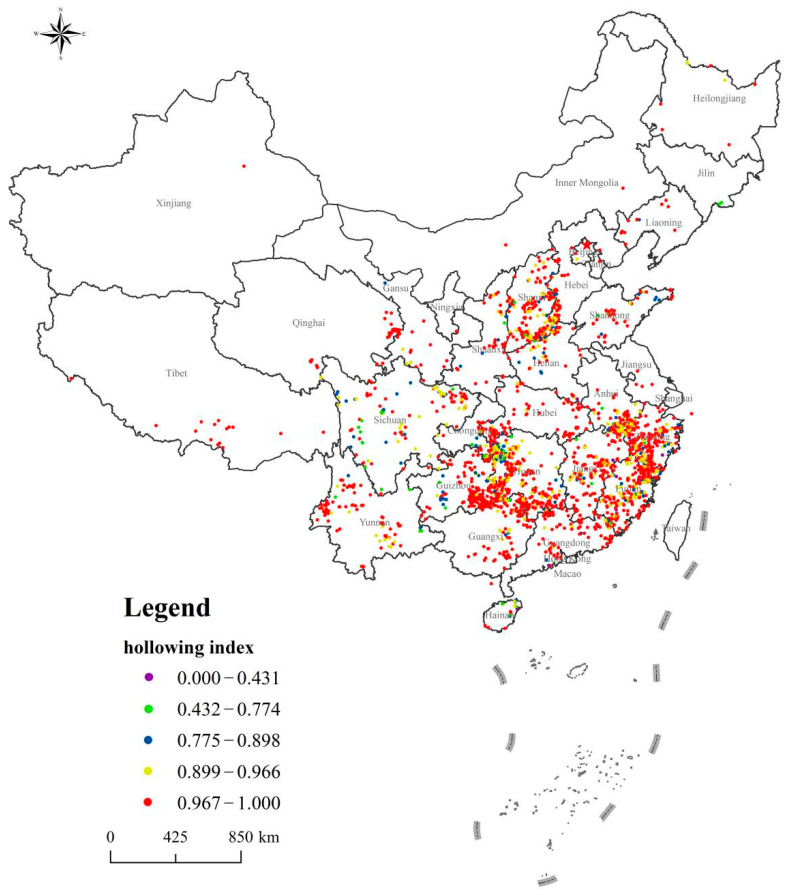
Distribution of Chinese traditional villages building hollowing index.

The mean value of the building hollowing coefficient for the 2645 traditional villages was 0.959. The median was 0.982, the standard deviation was 0.071, and the variance was 0.005. In general, the building hollowing degree was high, with more than 99% of traditional villages having a building hollowing index greater than 0.4 (see Figure 7). In terms of the per capita number of traditional buildings, more than 92% of the villages had a per capita number below 0.1. The per capita number was even smaller for the number of cultural relics and historical buildings.

In most traditional villages, there are basically no cultural relics or historical buildings. Most of the existing traditional buildings are idle, dilapidated, and on the verge of collapse. Chinese traditional buildings and cultural relics are mostly civil structures. Due to natural wear and tear from environmental factors (e.g., wind and rain) and poor maintenance, many historical buildings suffer significant damage and deterioration and eventually decay and collapse. Since many of these buildings are idle, uninhabited, and unattended, many cultural and historical buildings are in an accelerated aging process, hastening their descent to decay and disrepair. Moreover, given the accelerated levels of urbanization and modernization and associated human activities (e.g., “demolition of the old and the construction of a new one”), some traditional buildings and cultural relics are also threatened by being replaced and destroyed. In general, the combined effects of natural and anthropogenic factors exacerbate the degree of hollowing of traditional Chinese village buildings (see Figure 2).

### 4.5. Cultural Hollowing

**Figure 8:**
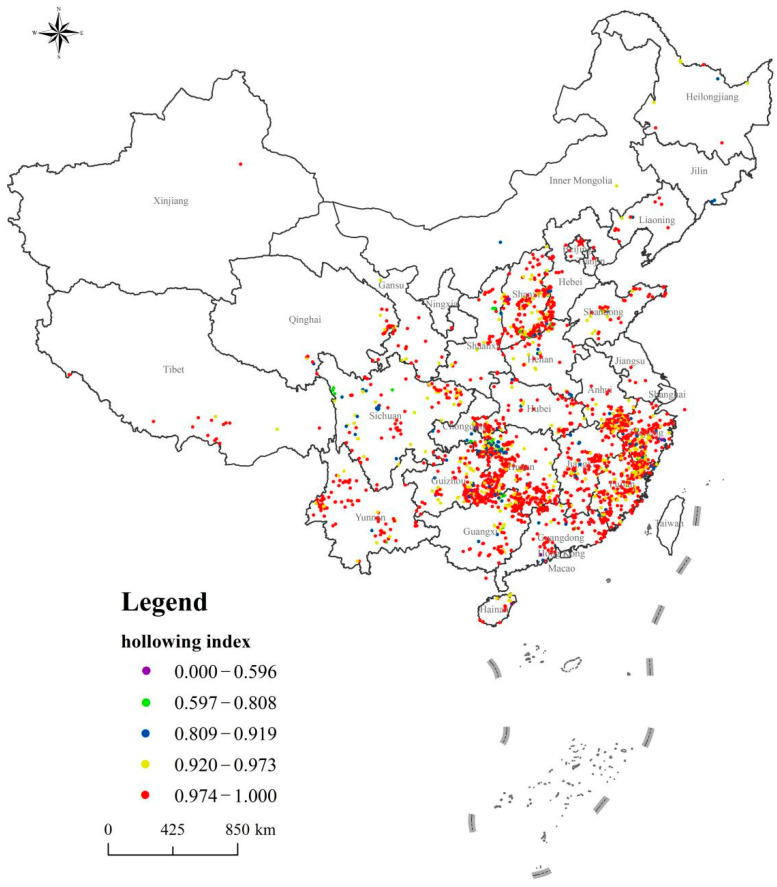
Distribution of Chinese traditional villages cultural hollowing index.

Due to population shifts coupled with the idleness of houses and public facilities, the traditional lifestyle, culture, and other aspects of traditional village living face obsolescence and extinction. The mean value of the cultural hollowing was 0.969, and the median was 0.987. The standard deviation was 0.058, the variance was 0.003, and the mode was 1 (the frequency of occurrence was 20%). In general, the cultural hollowing rate for most traditional villages was very high, and the cultural hollowing index of more than 99% of traditional villages was greater than 0.7 (see Figure 8). There were very few representative intangible cultural heritage projects in traditional villages at the national, provincial, and county levels. The few remaining representative projects of intangible cultural heritage have gradually evolved into “programs”, which do not fully protect and preserve the culture and heritage of traditional village life.

Representative projects of intangible cultural heritage are transmitted from one generation to the next. Since people who stay in traditional villages are mostly vulnerable groups, such as the elderly and children, they are unable to pass on the inheritance of representative intangible cultural heritage projects. Moreover, it requires a considerable workforce and material resources to transmit and inherit representative intangible cultural heritage projects, which makes finding a loyal inheritor extremely difficult.

## 5. Discussion

The hollowing of traditional villages is an interdisciplinary research topic in rural development and settlement geography. Unlike the general research on hollowing villages, the hollowing of traditional villages is a comprehensive topic involving rural economic development, social and cultural heritage, and regional sustainable development.

From a macro perspective, this study analyzed 2645 villages from the Chinese traditional village catalogue (Batch V) by the Ministry of Housing and Urban-Rural Development of the People’s Republic of China (MOHURD). Using a systematic analysis of population, architecture, cultural relics, and intangible cultural heritage, a hollowing evaluation index system was constructed. Through quantitative calculations on population hollowing, cultural hollowing, and building hollowing, the results suggest that the traditional villages have entered the stage of high hollowing. In our field observations and interviews, we also obtained similar conclusions. More than 90% of the respondents believe that the traditional villages around them have entered the stage of high hollowing, especially in hollowing population, buildings, and culture.

### 5.1. Influencing Factors and Mechanism

**Figure 9:**
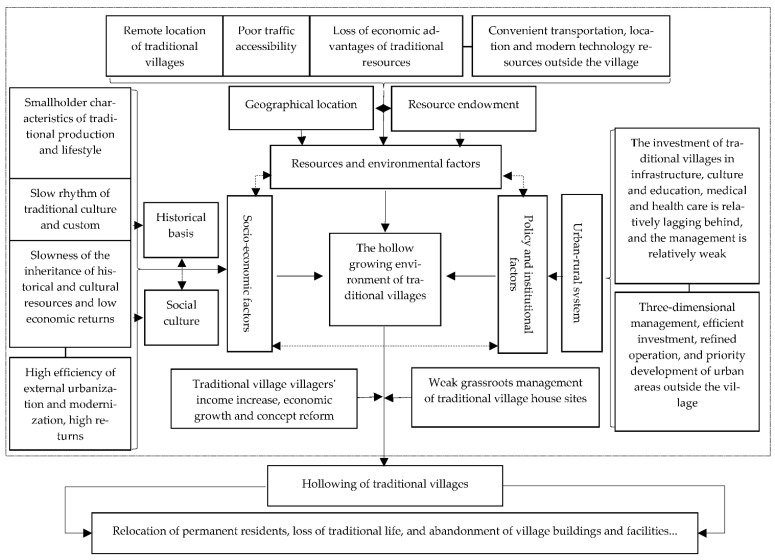
Influencing factors and mechanism framework of traditional villages hollowing.

The hollow growth environment of traditional villages can be regarded as a comprehensive model composed of economic, natural, social, cultural, institutional, and management factors [43,45,49,54]. Using the methods of expert consultation and summarizing applied in the Edraw Max software, we drew the influencing factors and mechanism framework of traditional villages hollowing (see Figure 9). Resource endowment and geographical locations are resource and environmental incentives for the hollowing of traditional villages. Historical basis and culture are the social and economic incentives. The urban–rural dual system is the external institutional reason. There is a significant non-linear interaction between these different dimensional factors. The hollow growth environment can only provide the prospect for the hollowing of traditional villages. Before such a possibility is realized, there must be a particular impetus driving the change, such as population shifts, technological development, and biological–natural factors. System reforms, policy support, technological advancement, employment transition, and other factors can accelerate the economic growth of villages and increase farmers’ income, resulting in significant changes to the traditional village lifestyle [6,7,8].

In terms of resources and environment, most Chinese traditional villages are located in remote mountainous or hilly areas. These areas generally have low accessibility, relatively poor transportation systems, and lagging scientific and technological resources. Over time, the original resource environment and economic advantages of traditional villages are gradually lost, and many traditional resources and endowments that traditional villages rely on for survival have disappeared. This makes maintaining the traditional production and lifestyle extremely difficult, and the unique attractiveness of traditional production and living is lost [17,18,19,20,21].

In socio-economic terms, traditional production methods and living concepts of traditional villages have extremely important values [47,48,49]. However, given its slow pace and low return rates, traditional production and lifestyle do not necessarily meet people’s modern and diversified needs for modern education, medical care, health, and entertainment. In addition, traditional culture, customs, and historical/cultural resources are inherited at a slow pace and have low economic returns. These hinder the inherent needs of traditional village economic development and income increase. Given the combined effects of the socio-economic development among traditional villages (slow effect, low return) and the external attraction of urbanization and modernization (high efficiency, fast return), the concept of production and life among traditional villages change, and their socio-economic system gradually disintegrates and becomes hollow [23,24,25,26,27,28].

Investments in infrastructure construction, cultural/educational resources, and healthcare among traditional villages lag behind their urban counterparts, and the management is relatively weak. Due to the increasing demands for new residential buildings, traditional houses and public buildings are being replaced and destroyed. Moreover, external forces (e.g., natural environment) significantly hinder the sustainable development of the material resources in traditional villages, resulting in the phenomenon of hollowing [36,37,38].

Moreover, other latent factors may also affect the hollowing of traditional villages. For example, the miniaturization of household-scale results in more disputes regarding the inheritance of old houses, causing complications in property rights and promoting the further hollowing of traditional villages. The low propensity to save the land, coupled with a weak concept of the legal system, can also lead to misperception of the value of traditional houses and public buildings and prompt their unrestricted use. Historical emotions and nostalgia for old houses and the influence of feudal superstitions such as “feng shui” could have also played a role in the formation of the hollow traditional village [44]. In addition, many residents who have economically progressed return to their hometowns and build houses in the countryside. However, since these houses are not used as primary residences, people are away for a long time, and their communities still suffer some degree of hollowing [43,45,49,54].

### 5.2. Recommendations

Given the current situation and potential impact of traditional village hollowing, developing an up-to-date and comprehensive catalogue of traditional villages is extremely important. It can provide policy support and protection of traditional villages and help enhance the attractiveness of traditional villages.

Additional measures should be taken to guide and regulate the hollowing of traditional villages. First, we should protect traditional village buildings at multiple levels. The protection of traditional buildings is not just about doing repairs, but more importantly, it is to retain their “popularity” and conduct regular care and management work [15,19]. We should also increase support for the development of traditional villages, invest in infrastructure and public services, strengthen traditional, cultural, and creative industries, and provide assistance and promotion to traditional village products [27,29]. We should also systematically plan the protection and inheritance of cultural resources in traditional villages, use modern technology to protect and preserve the inheritance of traditional cultural resources, increase training for heritage inheritors, and improve their sustainable viability [23,34,51].

Moreover, we should activate traditional village cultural spaces and use them more frequently for cultural events, entertainment activities, and academic seminars. This would minimize the idleness and obsolescence of resources and enhance the value of these places [46,49,52]. In terms of land use and project construction, we should manage traditional village resources more carefully [43,51,53]. The demolition of cultural buildings in traditional villages should be strictly prohibited, and measures should be put in place to protect cultural resources [30,40,46,47,48,49].

Rural restructuring is essential in developing regulations and measures addressing the hollowing of traditional villages. Rural reconstruction means the gradual rise of the rural service industry, the aggravation of population flow, and the renovation of the social and economic structure of rural areas [25,33,37,38,39]. The essence of rural reconstruction is to integrate and allocate material and non-material resources, including human, land, and financial capital and develop the rural economy, society, and spaces through reorganization, structural optimization, and functional evolution [24,36,37]. Policies, measures, and strategies addressing the hollowing of traditional villages in China should be guided to strengthen the hollowing rectification work, more precise policies should be issued, such as focusing on the cultivation of intangible cultural inheritors, focusing on the restoration of traditional buildings and historical buildings, and focusing on the development of characteristic industries [21,22,23,24,25,26].

Considering that 92% of all villages under investigation were classified as highly hollowed, targeted measures should be taken. Especially in capital input and policy support, different regions should take differentiated measures based on their own social and economic development conditions [33,34,35,36,37]. Each traditional village should implement measures based on its own resource endowment. In the future, given China’s rural revitalization policy, measures to promote the revitalization of hollowing traditional villages in the aspects of industrial development, population attraction, and landscape reconstruction should be further explored [50,51,52,53,54].

### 5.3. Conclusions

The hollowing of traditional villages is a complex and systematic work. It is a complicated and diverse economic and social phenomenon gradually formed by various factors. Due to data availability, this study focused on 2645 traditional Chinese villages, serving as an exploratory study on hollowing characteristics, geographical distribution, and influencing factors in terms of population, life, and facilities. Some indicators used in this study may not be suitable for the micro-scale analysis of traditional village hollowing, and some metrics suitable for micro-level may not be applicable for macro-scale. For example, the proportion of idle buildings may be more suitable for micro-level analysis. Determining which indicators to use at varying scales of analysis can be explored in future studies.

Land, capital, labor, and other elements of traditional villages will further agglomerate in cities and towns, putting increased pressure on traditional villages. The social economy and regional cultural system of traditional villages will further decline. Economic factors, such as the increase in farmer income and low land cost of building houses, significantly impact the hollowing of traditional villages. However, because achieving complete competition in the land market is difficult due to land use externalities, the formation mechanism of village hollowing cannot be fully defined based only on economics. Such market failure is more prominent in the hollowing of traditional villages, so it is necessary to include the influence of social group behavior, laws, regulations, and policies in understanding the hollowing of traditional villages. Analyzing the formation and evolution mechanism of hollowing of traditional villages has to be understood in terms of its evolution stage, considering the historical and cultural categories at different scales. Relevant influencing factors have to be decomposed into the different driving forces, such as social economy, policy system, resources, and environment, to better understand the progression of village hollowing. Comprehensive planning and administration should be undertaken to promote the sustainable development of the economy, society, and culture of traditional villages. Does the hollowing of traditional villages in each batch have similar attribute characteristics or spatial distribution? Can the indicators for the quantitative evaluation of traditional village hollowing be further optimized and adjusted for different research scales? Can more precise measures be taken for highly hollowed traditional villages? These questions can be further explored in future research.

## Figures and Tables

**Table 3:** Weighting results for the evaluation indexes of traditional villages hollowing.

Evaluation Index	Population Hollowing	Building Hollowing	Cultural Hollowing
Weighting result	0.30	0.43	0.27

**Table 4:** Chinese traditional villages hollowing degree.

Hollowing Index	Proportion	Main Provinces
(0.9, 1)	92.29%	Anhui, Beijing, Fujian, Gansu, Guangdong, Guangxi, Guizhou, Hainan, Hebei, Henan, Heilongjiang, Hubei, Hunan, Jilin, Jiangsu, Jiangxi, Liaoning, Inner Mongolia, Ningxia, Qinghai, Shandong, Shanxi, Shaanxi, Sichuan, Tianjin, Tibet, Xinjiang, Yunnan, Zhejiang, Chongqing
(0.8, 0.9)	6.31%	Anhui, Fujian, Guangdong, Guangxi, Guizhou, Hainan, Hebei, Henan, Hubei, Hunan, Jilin, Jiangxi, Liaoning, Shandong, Shanxi, Shaanxi, Sichuan, Yunnan, Zhejiang, Chongqing
(0.7, 0.8)	0.98%	Anhui, Fujian, Gansu, Guangdong, Guangxi, Guizhou, Hainan, Henan, Heilongjiang, Hunan, Jilin, Jiangxi, Liaoning, Shandong, Shanxi, Sichuan, Zhejiang, Chongqing
(0.6, 0.7)	0.30%	Fujian, Guangdong, Guangxi, Henan, Shanxi, Zhejiang
(0.5, 0.6)	0.11%	Hainan, Henan, Zhejiang
(0, 0.5)	0	None
